# Chromatin Condensation Delays Senescence in Human Mesenchymal Stem Cells by Safeguarding Nuclear Damages during *In Vitro* Expansion

**DOI:** 10.1155/2024/1543849

**Published:** 2024-05-10

**Authors:** Rohit Joshi, Tejas Suryawanshi, Sourav Mukherjee, Shobha Shukla, Abhijit Majumder

**Affiliations:** ^1^Department of Chemical Engineering, Indian Institute of Technology Bombay, Mumbai 400076, India; ^2^Centre for Research in Nano Technology and Science, Indian Institute of Technology Bombay, Mumbai 400076, India; ^3^Department of Metallurgical Engineering and Materials Science, Indian Institute of Technology Bombay, Mumbai 400076, India

## Abstract

Human mesenchymal stem cells (hMSCs) are multipotent cells that differentiate into adipocytes, chondrocytes, and osteoblasts. Owing to their differentiation potential, hMSCs are among the cells most frequently used for therapeutic applications in tissue engineering and regenerative medicine. However, the number of cells obtained through isolation alone is insufficient for hMSC-based therapies and basic research, which necessitates *in vitro* expansion. Conventionally, this is often performed on rigid surfaces such as tissue culture plates (TCPs). However, during *in vitro* expansion, hMSCs lose their proliferative ability and multilineage differentiation potential, rendering them unsuitable for clinical use. Although multiple approaches have been attempted to maintain hMSC stemness during prolonged expansion, finding a suitable culture system remains an unmet need. Recently, a few research groups have shown that hMSCs maintain their stemness over long passages when cultured on soft substrates. In addition, it has been shown that hMSCs cultured on soft substrates have more condensed chromatin and lower levels of histone acetylation compared to those cultured on stiff substrates. Furthermore, it has also been shown that condensing/decondensing chromatin by deacetylation/acetylation can delay replicative senescence in hMSCs during long-term expansion on TCPs. However, the mechanism by which chromatin condensation/decondensation influences nuclear morphology and DNA damage, which are strongly related to the onset of senescence, remains unknown. To answer this question, we cultured hMSCs for long duration in the presence of epigenetic modifiers, histone acetyltransferase inhibitor (HATi), which promotes chromatin condensation by preventing histone acetylation, and histone deacetylase inhibitor (HDACi), which promotes chromatin decondensation, and investigated their effects on various nuclear markers related to senescence. We found that consistent acetylation causes severe nuclear abnormalities, whereas chromatin condensation by deacetylation helps to safeguard the nucleus from damage caused by *in vitro* expansion.

## 1. Introduction

Human mesenchymal stem cells (hMSCs) are multipotent stem cells that can differentiate into various cell types, including adipocytes, chondrocytes, osteoblasts, and myoblasts. In addition, owing to their paracrine activity, hMSCs secrete a variety of cytokines and chemokines with immunomodulatory effects [1–3]. Owing to these excellent properties, hMSCs are an ideal cell source for tissue engineering and regenerative medicine (TERM) and are the most frequently used stem cells in clinical trials. Although the number of clinical studies has increased threefold over the last decade, very few studies have reached phase IV trials [4, 5]. While there are several interrelated issues that contribute to the paucity of late-phase trials, one is the lack of an effective method for *in vitro* expansion of hMSCs that retains their differentiation and therapeutic qualities. As the number of cells obtained through isolation alone is insufficient for basic research and/or therapeutic applications, hMSCs are expanded *in vitro*. In traditional scientific approaches, this is often performed by culturing hMSCs on rigid surfaces, such as tissue culture plates (TCPs). As for clinical applications, millions of cells per kilogram of patient body weight are required, the successful *in vitro* expansion of hMSCs is a prerequisite for their clinical application [6, 7]. However, during *in vitro* expansion, when hMSCs are expanded on TCPs, they enter the senescent state and lose their differentiation, secretory potential, migration, and homing ability, making them unsuitable for clinical use [8–10]. Like any other primary somatic cell, after a few passages, hMSCs cultured on TCPs undergo progressive decline in fitness as they enter the senescence state, which is morphologically characterized by enhanced spreading area and shape irregularity [11]. This decrease health of hMSC populations seen is likely due to unphysiologically rigid stiffness of tissue culture Petri plastics. Thus, there is a need for culture strategies that can maintain hMSCs fitness and potency during their long-term expansion. Although multiple approaches have been attempted to maintain MSC stemness during prolonged expansion, finding an easy-to-use culture system to achieve this is still an unmet need [12]. Studies have shown that mesenchymal stem cell proliferation, differentiation, and function are dependent on the extracellular mechanical environment including substrate stiffness [13–16]. Stiff substrate promotes osteogenic lineage and induces decreased expression of cell surface markers essential for hMSC function, while softer substrate promotes adipogenic lineage and retains regenerative properties [17–19]. Recently, a few research groups have shown that hMSCs maintain their stemness over long passages when cultured on an optimally soft polyacrylamide (PAA) gel, maintaining their cellular morphology and proliferative potential with delayed senescence [17, 18]. Furthermore, previous studies have also shown that mechanical cues such as substrate stiffness influence chromatin remodeling and epigenome of hMSCs regulating cellular behavior and fate [20–23]. Chromatin remodeling primarily regulates gene expression through epigenetic modifications, such as acetylation, methylation, and phosphorylation. The acetylation landscape is highly dynamic and governed by two classes of enzymes, histone acetyltransferases (HATs) and histone deacetylases (HDACs). The acetylation of histones by HATs leads to chromatin decondensation, which enables gene expression. Alternatively, histone deacetylation by HDACs results in condensation of chromatin and repression of gene expression. Recently, it has been shown that substrate stiffness regulates chromatin condensation, and hMSCs cultured on stiff substrates have more decondensed chromatin with higher histone acetylation than on soft substrates [20, 24].

As discussed previously, it is now known that replicative senescence in hMSCs can be delayed by culturing them on soft substrate, and soft substrate promotes chromatin condensation. Motivated by these findings, we hypothesized that changes in the epigenetic landscape (acetylation/deacetylation) of hMSCs during long-term expansion play a role in regulating senescence. Therefore, we sought to investigate how culturing hMSCs on TCPs for long-term expansion in the presence of epigenetic modifiers HATi (promotes chromatin condensation) and HDACi (promotes chromatin decondensation) influences hMSCs senescence. We cultured hMSCs in the presence of anacardic acid (HATi) and valproic acid (HDACi) during serial passing (P4–P11) on TCPS. Our results suggest that culturing hMSCs in the presence of anacardic acid delays cellular senescence by safeguarding nuclear morphology as compared to hMSCs cultured in the presence of valproic acid and control. The results of this study will help to identify chromatin-based critical parameters for the expansion of hMSCs with high therapeutic and regenerative potency, in addition to material-based strategies.

## 2. Material and Methods

### 2.1. Cell Culture

Bone-marrow-derived hMSCs were purchased from Lonza (Cat. No. #PT-2501). hMSCs were cultured in low-glucose DMEM (Himedia, AL006) supplemented with 16% FBS (Himedia, RM9955), 1% antibiotic-antimycotic (Himedia, A002), and 1% Glutamax (Gibco, 35050) under humidified conditions at 37°C with 5% CO_2_. The cells were trypsinized using TrypLE™ (Gibco, 12604021) once they reached 70% confluency.

### 2.2. Treatment with HDACi and HATi

To check the effect of HDACi and HATi, 0.5 mM of valproic acid (PHR 1061) and 30 *µ*M of anacardic acid (ANA) were added to the growth media after 24 hrs of seeding. For the differentiation experiments, VA and ANA were added to the differentiation media.

### 2.3. Differentiation Assays

hMSCs were seeded at 2000 cells/cm^2^ in a 12-well culture plate in growth medium for 24 h followed by differentiation media. Adipogenic (A10410; Invitrogen) and osteogenic (A10069; Invitrogen) differentiation kits were used in this study. Cells were incubated for 9 days in adipo- and 14 days in osteoinduction media before quantitative assays. The differentiation medium was changed every third day. After differentiation, the cells were fixed with 4% paraformaldehyde (PFA) for 30 min at room temperature, followed by staining with Oil Red O (Sigma, O0625) for adipogenic differentiation. After incubating with the staining solution for 20 min, the samples were washed three times with DPBS. Images were captured for quantitative analysis using an EVOS inverted microscope (Invitrogen) in a bright-field color channel.

### 2.4. Immunofluorescence Staining

The cells were fixed with 4% PFA in PBS for 15 min at room temperature (RT) and then washed three times with PBS. The cells were then permeabilized with permeabilizing buffer (0.5% Triton X-100, Sigma-Aldrich) for 10 min and blocked with BSA (4% bovine serum albumin in PBS) for 30 min to minimize nonspecific protein binding. Antilamin A (1 : 500; mouse; Abcam, Cat. No. 8980), antilamin B (1 : 500; rabbit; Abcam, Cat. No. 16048), and anti-AcK (1 : 500; rabbit; Abcam, Cat. No. 190479) primary antibodies in 4% bovine serum albumin (BSA) were added to the samples and incubated overnight at 4°C. The primary antibodies were removed, and the samples were rinsed twice with PBS for 10 min. The samples were then incubated at room temperature with secondary antibodies: donkey anti-mouse (1 : 500; Alexa Fluor 488, Cat. No. 21202), donkey anti-rabbit (Alexa Fluor 569, Cat. No. ab175470), phalloidin (1 : 400; Alexa Flour 532, Cat. No. A22282), and Hoechst 33342 (Cat. No. H3570) at a dilution of 1 : 5000 in 4% bovine serum albumin (BSA) for 2 h at room temperature. The samples were then rinsed twice with PBS. All immunostained samples were stored in PBS at 4°C until imaging was performed. Positive and negative controls were kept during immunofluorescence staining. All the samples were imaged at 63X (oil) magnification using a laser scanning confocal microscope (LSM, Carl Zeiss).

### 2.5. Quantification of Cell Morphology

Cell images were captured at different passages 48 h postseeding using an EVOS-FL auto-inverted microscope (Life Technologies) at 10x magnification. The cell spreading area was determined using ImageJ software (National Institutes of Health) by manually tracing the perimeter of individual cells. For each sample, a minimum of 100 random cells were analyzed. The number of protrusions of cells was manually quantified from phase-contrast images using ImageJ software.

### 2.6. BrdU Assay

To determine the percentage of S-phase cells in the cell cycle, cells from EP (early passage) and LP (late passage) were trypsinized and seeded on glass coverslips. After 48 h of seeding, BrdU reagent (Invitrogen, 000103) was added at a 1 : 100 (v/v) ratio to the media and incubated for 4 h at 37°C in a humidified incubator with 5% CO2. Thereafter, cells were fixed (4% paraformaldehyde), permeabilized (0.5% Triton-X), denatured (2 M HCL), blocked (1.5% bovine serum albumin), and incubated with anti-BrdU antibody (Invitrogen, B35128, 1 : 100) and counterstained with Alexa Fluor 568 (Invitrogen, A11061, 1 : 400). Immunofluorescence images were captured using EVOS-FL auto, and BrdU-positive and BrdU-negative cells were counted manually using the ImageJ software.

### 2.7. Senescence Assays

Senescence-associated *β*-galactosidase (SA-*β*-gal) was used to detect MSCs senescence using an SA-*β*-gal staining kit (Abcam, AB65351) according to the manufacturer's instructions. Briefly, EP and LP cells were seeded in a six-well plate and incubated in growth media. Cells were fixed, stained with *β*-gal solution, and incubated at 37°C without CO_2_. Ten to fifteen random images were captured for each condition for analysis using an EVOS inverted microscope (Invitrogen) in a bright-field color channel.

### 2.8. Statistical Analysis

Data are presented as the mean ± SEM (otherwise mentioned) and were analyzed using Microsoft Excel. The data were plotted using the GraphPad Prism. Differences were considered statistically significant at ^*∗*^*p* < 0.05, ^*∗∗*^*p* < 0.01, ^*∗∗∗*^*p* < 0.001, ns = nonsignificant, using Student's *t*-test, or a one-way or two-way ANOVA.

## 3. Results

### 3.1. HATi Increases Chromatin Condensation of hMSCs during Long-Term Expansion

**Figure 1:**
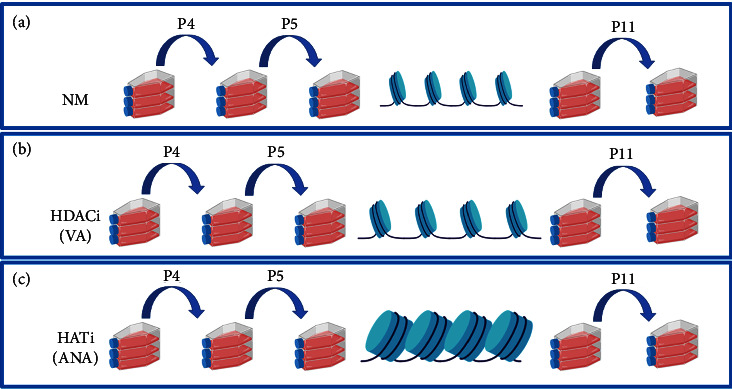
Schematic diagram showing the experimental workflow: Serial passaging of hMSCs was performed in TCPs from P4 to P11 cultured in (a) control (NM), (b) in the presence of VA, and (c) in the presence of ANA.

**Figure 2:**
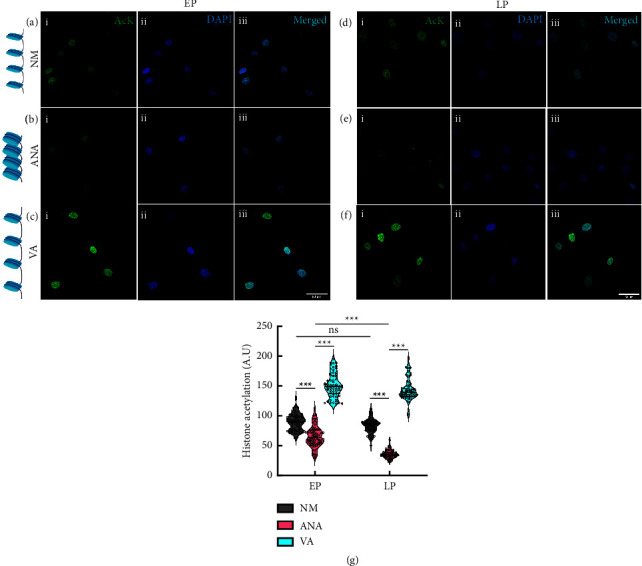
Staining of (i) global histone acetylation (Ack); (ii) DAPI; and (iii) merged image of nuclei when the cells were in ((a) EP and (d) LP) control (NM, without any inhibitor), ((b) EP and (e) LP) growth media with anacardic acid (HATi), and ((c) EP and (f) LP) growth media with valproic acid (HDACi). Blue: DAPI; Green, AcK; Cyan, merged. (g) Graph comparing histone acetylation under various conditions. (NM, normal media; ANA, anacardic acid; VA, valproic acid; *n* > 60 nuclei, three independent samples; scale bar = 50 *µ*m ^*∗*^*p* < 0.05, ^*∗∗*^*p* < 0.01, ^*∗∗∗*^*p* < 0.001, ns = nonsignificant.

Epigenetic modifications of histones are associated with senescence in various species and cells [25]. Histone acetylation is one of such critical epigenetic modifications. Here, we sought to investigate how a change in the global acetylation state of histones may influence replicative senescence during long-term *in vitro* expansion of hMSCs. To this end, we cultured hMSCs for multiple passages (P4–P11) in the presence of valproic acid (VA) and anacardic acid (ANA), two well-established inhibitors of histone acetylation (HDACi) and histone deacetylation (HATi), respectively (Figure 1). While HDACi keeps the chromatin in an open state by hyperacetylating the histones (Figure 1(b)), HATi maintained it in a more condensed state by inhibiting the acetylation process (Figure 1(c)). Our results showed that for early passages with respect to (normal media) NM (Figure 2(a)), there was a significant decrease in global histone acetylation in hMSCs cultured in the presence of ANA (∼25%) (Figures 2(b) and 2(g)) and a significant increase in the presence of VA (∼70%) (Figures 2(c) and 2(g)). Furthermore, when we cultured hMSCs from P4 to P11, we found that in late passages, there was no significant difference in histone acetylation for NM (Figures 2(d) and 2(g)) compared with the early passage. When cultured in the presence of the inhibitors HATi and HDACi, we found that in the late passage, there was a significant decrease in histone acetylation in the presence of ANA (∼45%) (Figures 2(e) and 2(g)), and no difference was observed when the cells were cultured with VA (Figures 2(f) and 2(g)) compared to the early passages.

### 3.2. Chromatin Condensation Helps in Maintaining hMSCs Proliferation during Long-Term Expansion

**Figure 3:**
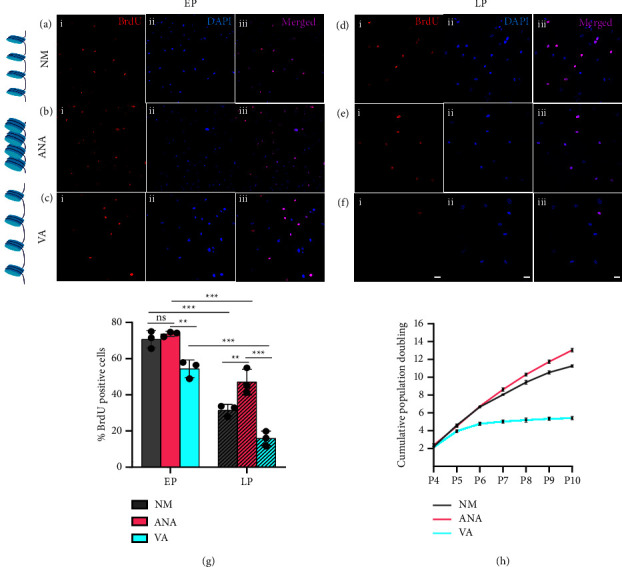
BrdU incorporation study to estimate proliferation of early passage (EP) (a)–(c) and late passage (LP) (d)–(f) for hMSCs cultured in normal media (NM) (a) and (d), with anacardic acid (ANA) (b) and (e), and with valproic acid (VA) (c) and (f). Blue: DAPI; red, nuclei with BrdU; magenta, merged. (g) Graph comparing BrdU incorporation under various conditions; (h) graph showing cumulative population doubling of hMSCs cultured under all three conditions (*n* > 400 nuclei, four independent samples, scale bar = 50 *µ*m, ^*∗*^*p* < 0.05, ^*∗∗*^*p* < 0.01, ^*∗∗∗*^*p* < 0.001, ns = nonsignificant).

Next, we investigated the effects of chromatin condensation and decondensation on the proliferation of hMSCs. The BrdU incorporation assay was used to identify proliferating cells. Our results showed that in the early passages, there was no significant difference in hMSCs proliferation between NM (Figures 3(a) and 3(g)) and ANA (Figures 3(b) and 3(g)). However, a significant decrease was observed in the presence of VA (∼24%) (Figures 3(c) and 3(g)) compared to NM, showing that hyperacetylation of histones by HDACi (VA) negatively influences the cell cycle. When hMSCs were cultured on TCPs for a long period, there was a significant decrease in proliferation in all three cases of LP compared to EP. In the late passages, hMSCs cultured in the presence of NM showed a significant decrease in proliferation (∼55%) (Figures 3(d) and 3(g)) compared with the early passage. When cultured in the presence of the inhibitors HATi and HDACi, ANA showed a minimal decrease in proliferation (∼33%) (Figures 3(e) and 3(g)), whereas VA showed the maximum decrease in proliferation (∼70%) (Figures 3(f) and 3(g)) in the LP than in the EP. Moreover, we checked for cumulative population doubling (CPD) for hMSCs cultured in all three conditions and found that cells cultured in the presence of ANA grew almost linearly up to P9, whereas CPD declined drastically for cells cultured in VA from P6 (Figure 3(h)), resulting in a difference in population doubling between ANA and VA at P10 of ∼8. In other words, culturing hMSCs in the presence of ANA would result in 2^8^ (256) times more cells than culturing in VA.

### 3.3. Chromatin Compaction Delays hMSCs Senescence

**Figure 4:**
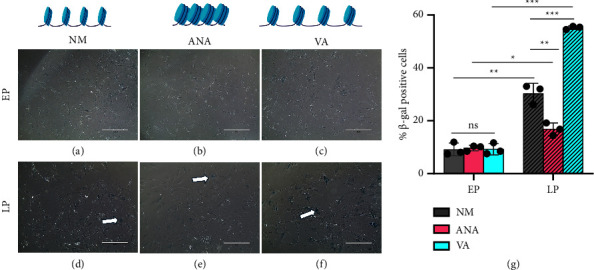
Investigation of senescence in early passage (EP) (a)–(c) and late passage (LP) (d)–(f) in the presence of normal media (NM) (a) and (d) with anacardic acid (ANA) (b) and (e) and valproic acid (VA) (c) and (f). (g) Percentage of *β*-gal-positive cells under various conditions (NM, ANA, and VA) showing the highest *β*-gal activity in hMSCs cultured in the presence of VA and lowest in the presence of ANA for late passage cells (*n* > 600 cells, four independent samples, scale bar = 400 *µ*m, ^*∗*^*p* < 0.05, ^*∗∗*^*p* < 0.01, ^*∗∗∗*^*p* < 0.001, ns = nonsignificant).

To check the effect of HDACi and HATi on hMSCs senescence, we examined *β*-gal activity, which is the gold standard assay to assess senescence. Our results showed that there was no significant difference in *β*-gal activity for all three conditions for hMSCs (NM, ANA, and VA) cultured during early passage (P4), as shown in Figures 4(a)–4(c) and 4(g). However, when we checked for late passage (P11), we found that there was a significant increase in *β*-gal activity under all three conditions, as shown in Figures 4(d)–4(f) and 4(g). When cultured in NM, a significant increase (∼3.3 times) (Figures 4(d) and 4(g)) in the LP group than in the EP group. When cultured in the presence of HATi and HDACi, cells cultured with ANA showed a minimal increase (∼1.5 times) (Figures 4(e) and 4(g)), and VA showed a maximum increase (∼5 times) (Figures 4(f) and 4(g)) in *β*-gal activity in LP compared to that in EP. Furthermore, we observed that the cells cultured in the presence of HDACi showed a drastic deterioration in cellular morphology in terms of cell spread area and number of protrusions as compared to NM and ANA (Figure S1) further supporting the senescent phenotype induced by HDACi treatment. In conclusion, our results showed that culturing hMSCs for long-term expansion in a condensed chromatin state in the presence of HATi delays hMSCs senescence.

### 3.4. Lamin A Expression Increases with Serial Passaging

**Figure 5:**
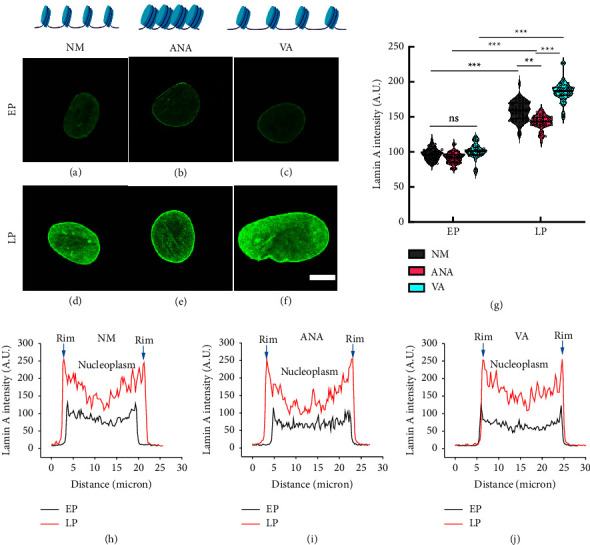
Immunofluorescence images of lamin A in early passage (EP) (a)–(c) and late passage (LP) (d)–(f) hMSCs cultured in normal media (NM) (a) and (d), with anacardic acid (ANA) (b) and (e)), and valproic acid (VA) (c) and (f). (g) Comparison of lamin A intensity under various conditions (NM, ANA, and VA); distribution of lamin A intensity across nuclei in (h) normal media, (i) presence of anacardic acid, and (j) presence of valproic acid (*n* > 60 nuclei, three independent samples, scale bar = 10 *µ*m, ^*∗*^*p* < 0.05, ^*∗∗*^*p* < 0.01, ^*∗∗∗*^*p* < 0.001, ns = nonsignificant).

Lamin expression levels are important senescence markers. As shown by earlier researchers, there is an accumulation of lamin A and loss of lamin B in the senescent cells. Therefore, we investigated the effects of chromatin decondensation (HDACi) and chromatin condensation (HATi) on lamin A expression during serial passaging. We observed that hMSCs cultured in the early passage (P4) had no significant difference in total lamin A intensity under all three conditions (NM, ANA, and VA) (Figures 5(a)–5(c) and 5(g)). Moreover, we found that in early passage cells, lamin A localized to the nuclear periphery rather than to the nucleoplasm, as shown in Figures 5(a)–5(c). Next, we investigated lamin A expression in late passage cells in the presence of all three conditions and found that there was a significant increase in lamin A expression in all three cases (NM, VA, and ANA) compared to that in early passage cells. We found that late passage cells cultured in NM showed a significant increase in lamin A expression (∼1.6 times) (Figures 5(d) and 5(g)) compared to early passage cells. When cultured in the presence of ANA and VA, cells in ANA showed a minimal increase (∼1.4 times) (Figures 5(e) and 5(g)), and VA showed the maximum increase (∼1.8 times) in lamin A expression in LP with respect to EP. From our results, we also found that in late passage cells, lamin A was not only localized in the nuclear periphery, but also accumulated in the nucleoplasm, as shown in Figures 5(h), 5(i), and 5(j). Furthermore, we examined the effects of HDACi (chromatin decondensation) and HATi (chromatin condensation) on lamin B expression during serial passaging. We observed that hMSCs cultured in the presence of HDACi showed a maximum decline in lamin B expression (Figure S2), whereas hMSCs cultured in the presence of HATi showed a minimal decline in lamin B expression (Figure S2), further supporting the hypothesis that HDACi induces senescence in hMSCs.

### 3.5. Chromatin Condensation Maintains Adipogenic Differentiation Potential during Long Expansion

**Figure 6:**
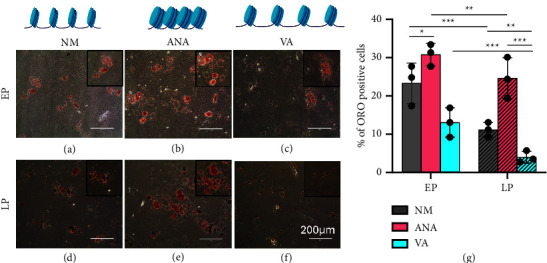
Investigation of adipogenic differentiation of hMSCs by Oil Red O staining on early passage hMSCs in (a) control (NM), (b) presence of anacardic acid, (c) presence of valproic acid and in late passage hMSCs in (d) control (NM), (e) presence of anacardic acid, and (f) presence of valproic acid, and (g) graph comparing percentage of Oil Red O-positive cells under various conditions (NM, normal media; ANA, anacardic acid; VA, valproic acid) (four independent samples, scale bar = 200 *µ*m, ^*∗*^*p* < 0.05, ^*∗∗*^*p* < 0.01, ^*∗∗∗*^*p* < 0.001, ns = nonsignificant).

Previous studies have shown that hMSCs differentiation ability is passage-dependent and adipogenic differentiation potential decreases with serial expansion [26]. Motivated by these findings, we investigated the effect of chromatin condensation/decondensation on the adipogenic differentiation potential of hMSCs cultured during serial passaging. Our results showed that with respect to NM (Figure 6(a)), there was a significant increase in the percentage of ORO-positive cells in the ANA (30%) (Figures 6(b) and 6(g)) and a significant reduction when cultured in the presence of VA (∼43%) (Figure 6(c) and 6(g)).

Furthermore, when we cultured hMSCs for a long period, there was a decline in adipogenic differentiation potential in all three cases in LP compared to EP (Figure 6(g)). We found that in late passages, hMSCs cultured in NM showed a significant decrease (∼56%) in adipogenic differentiation potential (Figures 6(d) and 6(g)) compared with the early passages. When cultured in the presence of HATi and HDACi, the cells cultured in the presence of ANA showed a minimum decline (∼23%) (Figures 6(e) and 6(g)), and VA showed the maximum decline (∼69%) (Figures 6(f) and 6(g)) in the LP than in the EP group.

Supporting our previous results, hMSCs cultured for a long period in the presence of ANA showed delayed senescence while maintaining adipogenic differentiation potential as compared to NM and VA. Overall, our findings demonstrate that culturing hMSCs during serial passaging in the presence of ANA decreases senescence and rescues the adipogenic differentiation potential.

### 3.6. Chromatin Condensation Safeguards Nuclear Morphology during Long-Term Expansion

**Figure 7:**
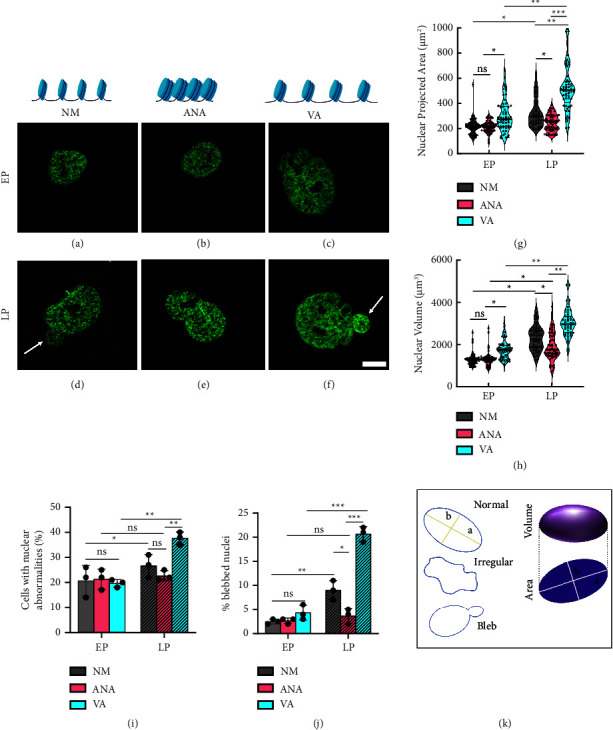
Chromatin condensation safeguards nuclear morphometric parameters during long-term expansion. Immunofluorescence images of acetylation showing nuclear blebbing in early passage (EP) (a)–(c) and late passage (LP) (d)–(f) for hMSCs cultured in normal media (NM) (a) and (d), with anacardic acid (ANA) (b) and (e), and with valproic acid (VA) (c) and (f). (g) Graph comparing change in nuclear-projected area, (h) volume, (i) percentage of cells with nuclear abnormalities, and (j) percentage of cells with nuclear bleb (k). Schematic showing abstract representation of normal, irregular, and blebbed nuclei (*n* > 65 nuclei, three independent samples, scale bar = 10 *µ*m ^*∗*^*p* < 0.05, ^*∗∗*^*p* < 0.01, ^*∗∗∗*^*p* < 0.001, ns = nonsignificant). Representative images (a)–(f) do not reflect the acetylation level as the intensities were modified to clearly show the nuclear boundaries.

It is now known that distorted nuclear morphology including abnormalities such as nuclear blebbing is strongly associated with cellular senescence [27, 28]. Senescent cells showed prominent nuclear blebs, which changed the overall 3D morphology of the nuclei. In the previous section, we demonstrated that cells cultured for multiple passages in the presence of ANA (deacetylation) exhibited lower *β*-gal activity, which is a standard marker for evaluating cellular senescence. However, the influence of persistent acetylation/deacetylation on the nuclear morphometric parameters during long-term expansion remains unexplored. To fill this gap, we investigated the effect of chromatin condensation/decondensation on nuclear morphometric parameters in hMSCs cultured on TCPs during serial passaging. We compared the early and late passage nuclear morphology and bleb and nuclear abnormalities in hMSCs cultured under all three conditions (NM, ANA, and VA). First, we analyzed the nuclear morphological parameters (projected area, volume, surface area, and circularity) and found that in the early passages, there was no significant difference between NM and ANA for any of the nuclear morphometric parameters (Figures 7(a), 7(b), 7(g), and 7(h)). However, there was a significant increase in the nuclear morphometric parameters for cells cultured in the presence of VA (area ∼40%, volume ∼34%, and surface area ∼34%) compared to NM (Figures 7(a), 7(c), 7(g), and 7(h)). Furthermore, when hMSCs were cultured for a long duration, there were significant differences in all nuclear morphometric parameters for all three conditions in LP compared to EP. During late passage, when cultured in the presence of NM, there was a significant increase in the projected nuclear area (∼59%, Figures 7(d) and 7(g)), volume (∼67%), and surface area (∼61%) compared to early passage. However, when cultured in the presence of HATi and HDACi, we found that hMSCs cultured in the presence of ANA showed a minimum increase in all nuclear morphometric parameters: nuclear-projected area (∼15%, Figure 7(g)), nuclear volume (∼44%; Figure 6(h)), and surface area (∼37%, Figure S3), but there was no significant change in circularity (Figure S3) in the LP than in the EP. When cultured in the presence of VA, the largest increase was observed in the projected nuclear area (∼72%, Figure 7(g)), volume (∼70%, Figure 7(h)), and surface area (∼60%, Figure S3), and a significant decrease in circularity (∼6%, Figure S3) in the LP than in the EP. Furthermore, we examined the effects of HDACi and HATi on nuclear blebbing and abnormalities. In the early passage, there was no significant difference in nuclear abnormalities among the three conditions (Figure 7(i)) and no significant difference in nuclear blebbing between NM and ANA; however, VA showed an approximately 80% increase in nuclear blebbing compared to NM (Figure 7(j)). Furthermore, when hMSCs were cultured for a long period, there was a significant increase in nuclear abnormalities (except ANA) and nuclear blebbing under all three conditions in LP compared with EP (Figures 7(a)–7(f), 7(i), and 7(j)). We showed that late passage cells cultured in NM showed a significant increase in nuclear abnormalities (∼32%) and blebbing (∼260%) (Figures 7(i) and 7(j)) than in early passages. When cultured in the presence of inhibitors HATi and HDACi, hMSCs cultured in the presence of ANA showed a minimal increase in nuclear abnormalities (∼11%) and nuclear blebs (∼133%) and maximum increases in nuclear abnormalities (∼97%) and blebs (∼377%, Figures 7(f) and 7(j)) when cultured in the presence of VA in LP compared to EP. Overall, our data suggest that long-term culture of hMSCs in the presence of HATi (ANA) prevents deterioration of nuclear morphology, nuclear blebs, and abnormalities, which are markers of cellular senescence and aging.

### 3.7. Chromatin Condensation Prevents DNA Damage during Long-Term Expansion

In our previous section, we demonstrated that culturing hMSCs during long-term expansion in the decondensed chromatin state (HDACi) deteriorates nuclear morphology while culturing them in condensed chromatin state in the presence of HATi safeguards it. Building on these findings, we next investigated the protective effect of chromatin compaction through HATi on DNA damage. It is well established that DNA strand break is one of the major triggers of cellular senescence leading to the activation of tumor suppressor proteins, such as p53 and p16, and can accelerate telomere shortening. Hence, to study the effect of chromatin condensation/decondensation on DNA damage, we stained for *γ*-H2AX, which is the most sensitive and common marker for double-stranded DNA breaks.

**Figure 8:**
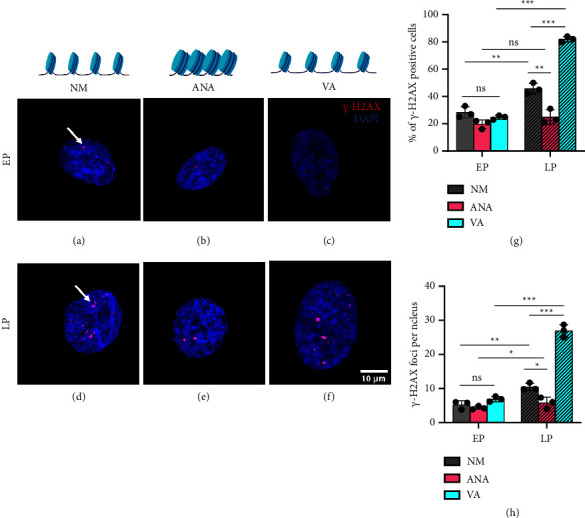
Chromatin condensation safeguards nuclei from double-stranded DNA breaks. Immunofluorescence images of *γ*-H2AX (red) and DAPI (blue) showing DNA damage in early passage (EP) (a)–(c) and late passage (LP) (d)–(f) for hMSCs cultured in normal media (NM) (a) and (d), with anacardic acid (ANA) (b) and (e), and valproic acid (VA) (c) and (f). (g and h) Graph comparing the percentage of *γ*-H2AX-positive cells and *γ*-H2AX foci per nucleus under various conditions (NM, normal media; ANA, anacardic acid; VA, valproic acid, *n* > 60 nuclei, three independent samples, scale bar = 50 *µ*m and 10 *µ*m (zoom-in), ^*∗*^*p* < 0.05, ^*∗∗*^*p* < 0.01, ^*∗∗∗*^*p* < 0.001, ns = nonsignificant).

As expected from previous studies, we also observed that the late passage cells cultured in NM showed a significant increase in *γ*-H2AX-positive cells (∼1.5 times) and *γ*-H2AX foci per nucleus (∼2 times) compared to their EP counterparts, re-establishing the link between replicative senescence and DNA damage (Figure 8). This increase was common for all the three conditions. However, while the increase was the most prominent for VA, the same was the least for ANA, indicating that HDACi favors DNA damage, whereas HATi safeguards against it. When cultured in the presence of ANA and VA, cells in ANA showed a minimum increase and VA showed the maximum increase in the percentage of *γ*-H2AX-positive cells in the LP (∼1.3 times for ANA and ∼3.3 times for VA) (Figure 8(g)) and in *γ*-H2AX foci (∼1.3 times for ANA and ∼4 times for VA) (Figure 8(h)). Altogether, our data show that cells cultured in the presence of HATi during long-term expansion maintain nuclear morphology and integrity and show minimal DNA damage, whereas cells cultured in the presence of HDACi show a drastic increase in DNA damage.

## 4. Discussion

Histone acetylation/deacetylation is a critical epigenetic modification that regulates gene expression by condensing or decondensing the chromatin. While these changes occur locally in the regulation of particular genes, global histone hyperacetylation has been reported to be associated with cellular senescence [29–31]. It has been demonstrated that hyperacetylation of histones (either by inhibiting HDACs or upregulating HATs) accelerates senescence, resulting in a reduced proliferation rate. In contrast, hypoacetylation (by HATi) maintains cell proliferation [32, 33]. Interestingly, *in vivo* studies using model organisms, such as Drosophila, Caenorhabditis elegans, and zebrafish, have shown an opposite trend [34–36]. Among the many differences between these *in vitro* and *in vivo* studies, one fundamental difference is that while *in vivo* cells experience the effect of cell division over multiple generations, most *in vitro* studies are performed for a single passage, with only one exception to the best of our knowledge. In the only available literature studying the effect of HDACi on long-term expansion of hMSCs, Jung et al. showed that HDACi induces cellular senescence during long-term expansion through downregulation of polycomb group genes (PcGs) and upregulation of Jumonji domain-containing protein 3 (JMJD3) [32]. The same study also demonstrated that condensing chromatin through HATi delayed hMSCs senescence by upregulating polycomb genes and maintaining proliferation. Clearly, the effect of histone acetylation and deacetylation on cellular senescence in long-term cultures requires further investigation.

Consistent with the study mentioned above, we found that hyperacetylation of histones with HDAC inhibitors accelerated senescence, while hypoacetylation with HAT inhibitors delayed senescence. We confirmed this observation using well-established markers of senescence, such as beta-galactosidase activity, cellular morphology, and nuclear lamin A expression, as well as by monitoring the population doubling time and proliferation rate. Previously, it has been shown that hMSCs aging is associated with the differential expression of different types of histone acetylation (e.g., decrease in H3K9 and H3K14 and increase in H3K56 acetylation) [37]. However, in our study, we checked for pan acetylation and found no significant difference between early and late passage cells in the presence of NM and HDACi. Furthermore, we observed that long-term passaging induced replicative senescence under all three conditions (control, HAT inhibitor ANA, and HDAC inhibitor VA), with the HAT inhibitor showing the lowest and the HDAC inhibitor showing the highest degree of senescence. The accumulation of lamin A is known to cause nuclear abnormalities that decrease DNA stability and DNA damage [38]. Our results show that HATi limits the accumulation of lamin A in late passage cells (Figure 5), which helps to prevent nuclear deformity (Figure 7) and DNA damage (Figure 8).

Interestingly, we observed a decline in hMSC proliferation in the presence of HDAC inhibitors in early passages, before any significant increase in *β*-galactosidase activity or lamin A expression. As senescence is a gradual and multistep decline in cellular physiological processes, we propose that the initial decline in proliferation is the first prominent senescence-induced outcome due to chromatin decondensation via HDAC inhibitors, among other senescence-associated phenotypes.

Another crucial issue in *in vitro* hMSC expansion is the gradual loss of differentiation potential with serial passaging. hMSCs have tri-lineage differentiation potential, making them a promising tool for tissue engineering and regenerative medicine. However, their adipogenic differentiation potential declines with long-term *in vitro* expansion [18]. Here, we have shown that when expanded in the presence of HAT inhibitors, the adipogenic differentiation potential of late passage cells matches that of early passage cells cultured without any inhibitor.

The findings of this study introduce a fascinating possibility that warrants further investigation. Previous research from our group has demonstrated that culturing cells on softer substrates can delay senescence and preserve the differentiation potential of hMSCs and keratinocytes [18, 39]. Moreover, multiple studies, including our own, have shown that chromatin remains in a condensed state when cells are cultured on a soft substrate, similar to the effect of HAT inhibitor treatment [20, 24]. Given that both of these conditions—culturing cells on soft substrates and treating them with HAT inhibitors—delay senescence and maintain differentiation potential, it is conceivable that substrate stiffness influences senescence through its impact on hMSC fate across multiple passaging events, by altering histone acetylation.

In addition to other manifestations of senescence, the deterioration of nuclear morphological integrity is critical. Research has established that the nuclei of aging cells undergo various detrimental transformations, such as enlargement, loss of shape integrity, and bleb formation, which contribute to senescence [28]. While studies on short-duration drug treatment have shown that HDAC inhibitors cause nuclear abnormalities, no long-term study has been conducted to investigate the effect of histone acetylation status on nuclear structure [40, 41]. We have shown that HDAC inhibitors cause nuclear abnormalities, whereas HAT inhibitors maintain the integrity of the nuclear architecture. HAT inhibitors also protect the nucleus from DNA damage during expansion. Our results suggest that HAT inhibitors delay senescence by protecting the nucleus against DNA damage, which is reflected by less blebbing and maintenance of nuclear shape.

In summary, our study suggests that the use of HAT inhibitors can delay hMSC senescence by preserving nuclear morphology, which is evidenced by reduced nuclear blebbing and irregularities during long-term expansion, in addition to other reduced senescence-related phenotypes and maintained proliferation. These findings pave the way for the development of a culture system that enables the expansion of hMSCs while maintaining their self-renewal capability and differentiation potential by delaying senescence.

## Data Availability

The data used to support the findings of this study are included within the article and supplementary information.

## Supplementary Materials

Supplementary Materials This file includes data regarding (a) cells cultured in the presence of HDACi showed a drastic deterioration in cellular morphology in terms of cell spread area and number of protrusions as compared to NM and ANA (Figure S1); (b) hMSCs cultured in the presence of HDACi showed a maximum decline in lamin B expression (Figure S2), whereas hMSCs cultured in the presence of HATi showed a minimal decline in lamin B expression; (c) comparison between nuclear surface area and nuclear circularity under various conditions (NM—normal media, ANA—anacardic acid, VA—valproic acid) between early and late passage (Figure S3). Supplementary Materials have been uploaded in a separate file.
