# Supplementary material for: Chromatin Condensation Delays Senescence in Human Mesenchymal Stem Cells by Safeguarding Nuclear Damages during In Vitro Expansion
Source: J Tissue Eng Regen Med. 2024 May 10;2024:1543849. doi: 10.1155/2024/1543849 (PMC11919206; doi:10.1155/2024/1543849)
Supplement: Supplementary Materials — This file includes data regarding (a) cells cultured in the presence of HDACi showed a drastic deterioration in cellular morphology in terms of cell spread area and number of protrusions as compared to NM and ANA (Figure S1); (b) hMSCs cultured in the presence of HDACi showed a maximum decline in lamin B expression (Figure S2), whereas hMSCs cultured in the presence of HATi showed a minimal decline in lamin B expression; (c) comparison between nuclear surface area and nuclear circularity under various conditions (NM—normal media, ANA—anacardic acid, VA—valproic acid) between early and late passage (Figure S3). Supplementary Materials have been uploaded in a separate file. [file 1543849.f1.zip › 1543849_supplementary_figure.pdf]

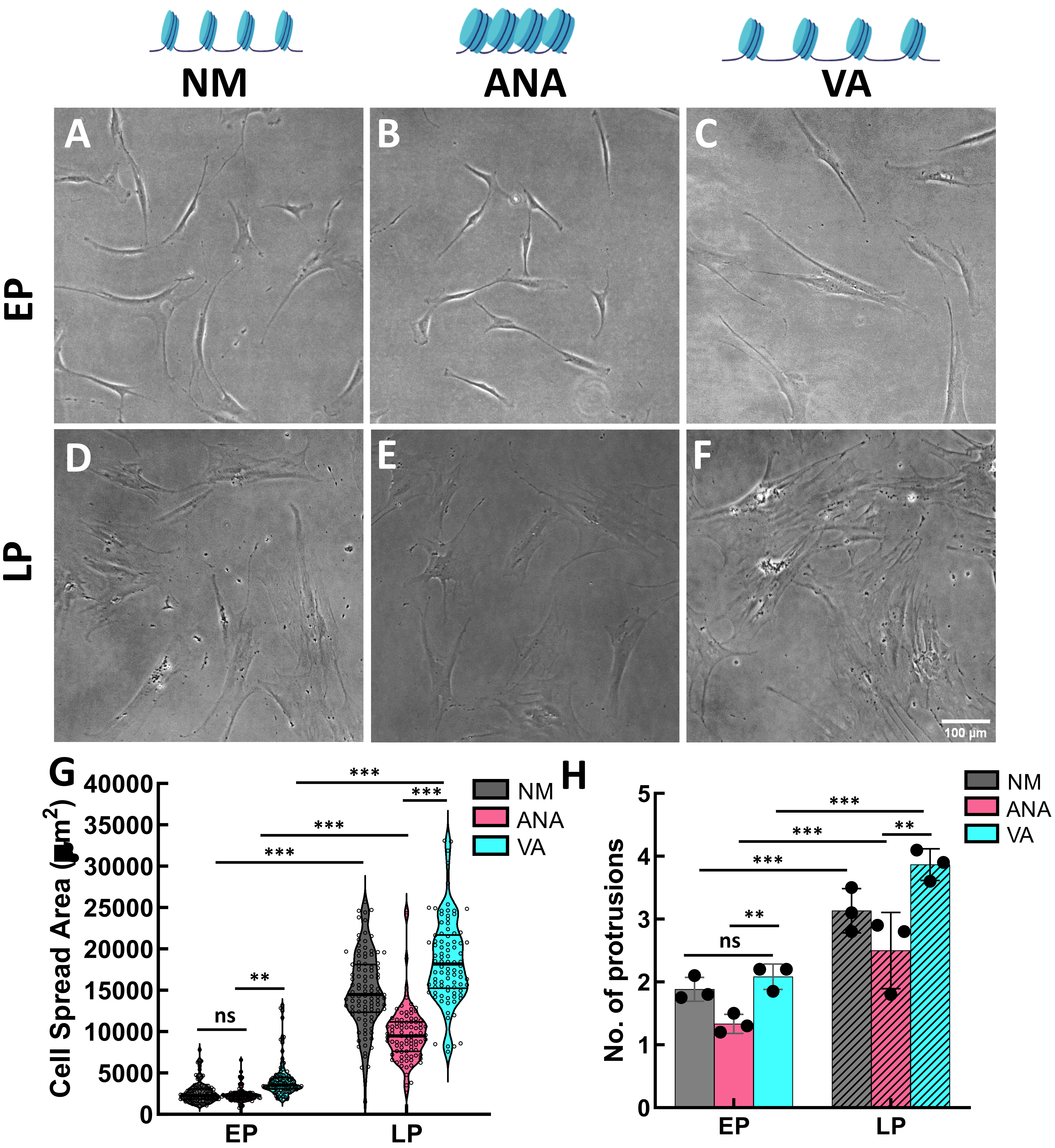

NM
ANA
VA
EP
LP
A
B
C
D
E
F
100 µm
G
Cell Spread Area (µm^2)
40000
35000
30000
25000
20000
15000
10000
5000
0
EP
LP
ns
**
***
NM
ANA
VA
H
No. of protrusions
5
4
3
2
1
0
EP
LP
ns
**
***
NM
ANA
VA

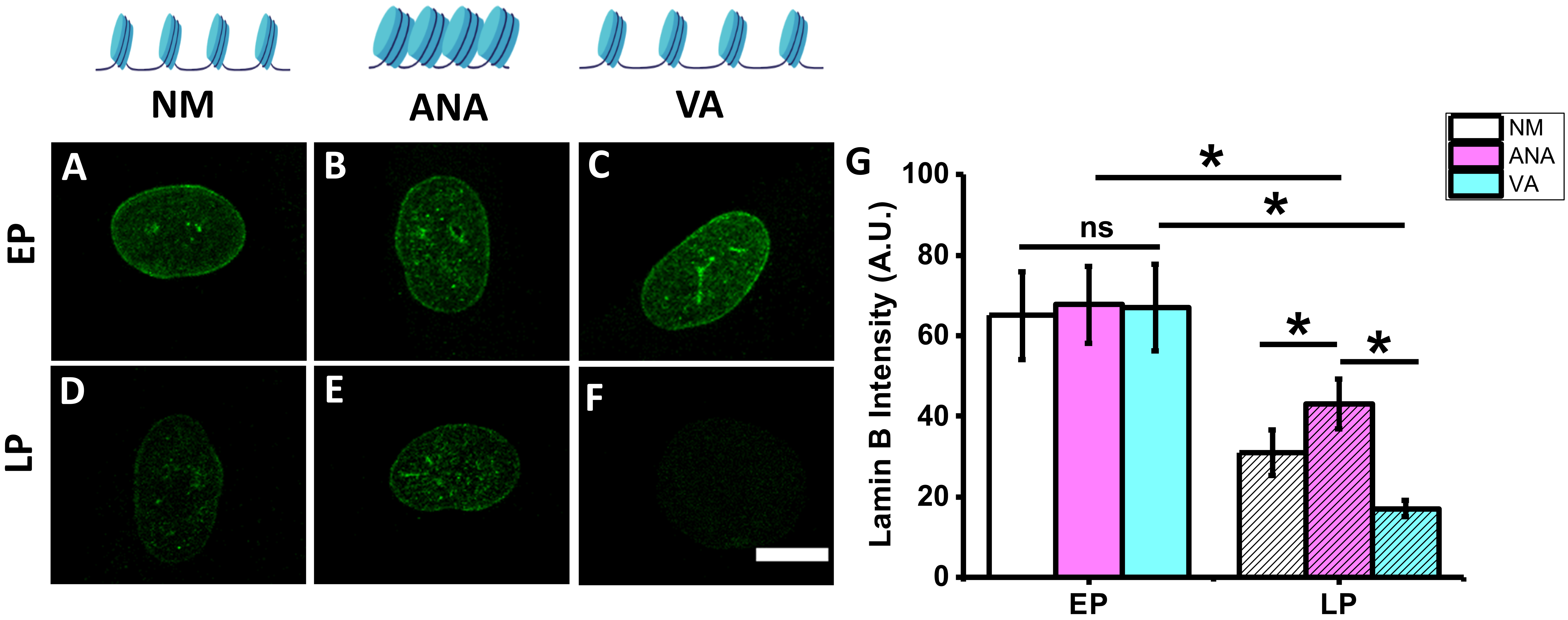

Figure S2. Immunofluorescence images of lamin B in early passage (EP, A-C) and late passage (LP, D-F) for hMSCs cultured in normal media (NM, A and D), with anacardic acid (ANA, B and E), and with valproic acid (VA, C and F). (G) Graph comparing lamin B intensity under various conditions (NM, ANA, VA), (NM- normal media, ANA- anacardic acid, VA – valproic acid); (n> 25 nuclei, 2 independent samples, Scale bar = 10 μm, *p<0.05, ns = non-significant).

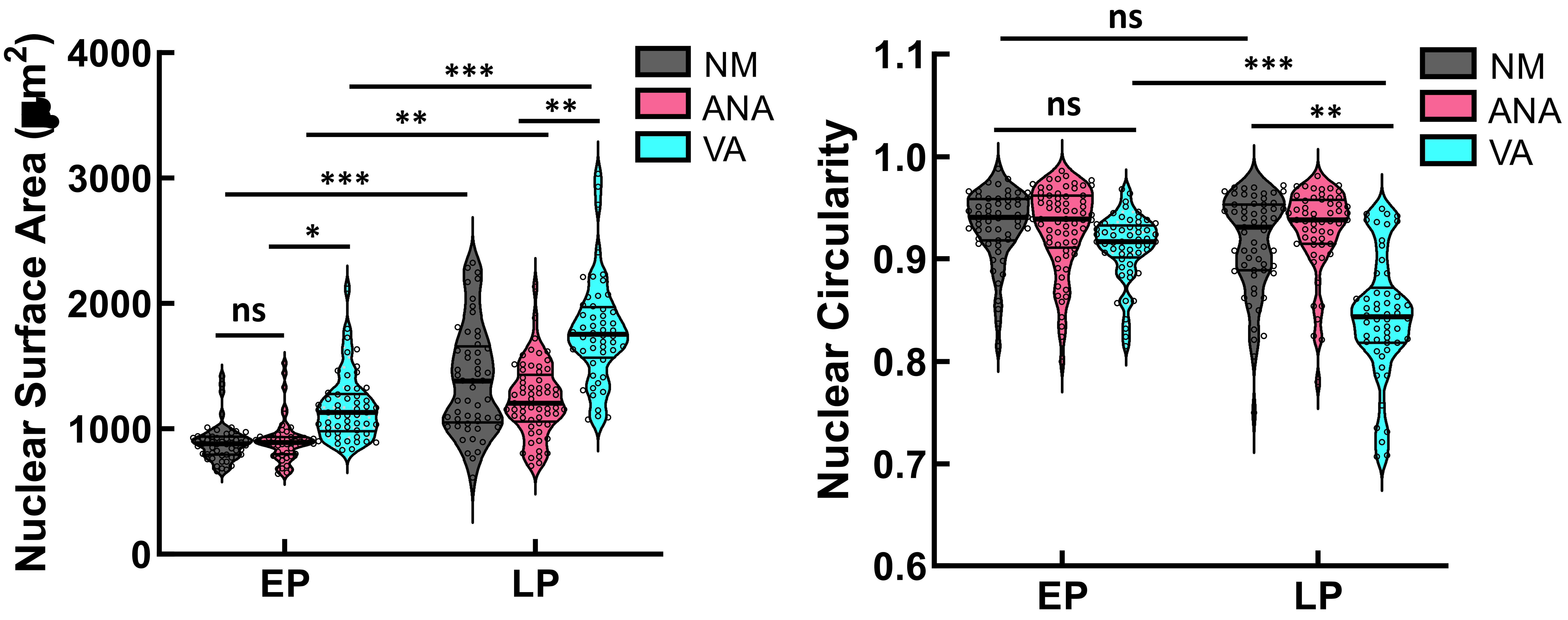

Figure S3. Graph comparing nuclear surface area and nuclear circularity under various conditions (NM- normal media, ANA- anacardic acid, VA- valproic acid) between early and late passage (n> 50 nuclei, 3 independent samples, Scale bar = 10 µm, *p<0.05, *p<0.05, **p<0.01, ***p<0.001, ns = non-significant).
