# Supplementary material for: Chromatin Condensation Delays Senescence in Human Mesenchymal Stem Cells by Safeguarding Nuclear Damages during In Vitro Expansion
Source: J Tissue Eng Regen Med. 2024 May 10;2024:1543849. doi: 10.1155/2024/1543849 (PMC11919206; doi:10.1155/2024/1543849)
Supplement: Supplementary Materials — This file includes data regarding (a) cells cultured in the presence of HDACi showed a drastic deterioration in cellular morphology in terms of cell spread area and number of protrusions as compared to NM and ANA (Figure S1); (b) hMSCs cultured in the presence of HDACi showed a maximum decline in lamin B expression (Figure S2), whereas hMSCs cultured in the presence of HATi showed a minimal decline in lamin B expression; (c) comparison between nuclear surface area and nuclear circularity under various conditions (NM—normal media, ANA—anacardic acid, VA—valproic acid) between early and late passage (Figure S3). Supplementary Materials have been uploaded in a separate file. [file 1543849.f1.zip › Supplementary_material .docx]

**Supplementary**


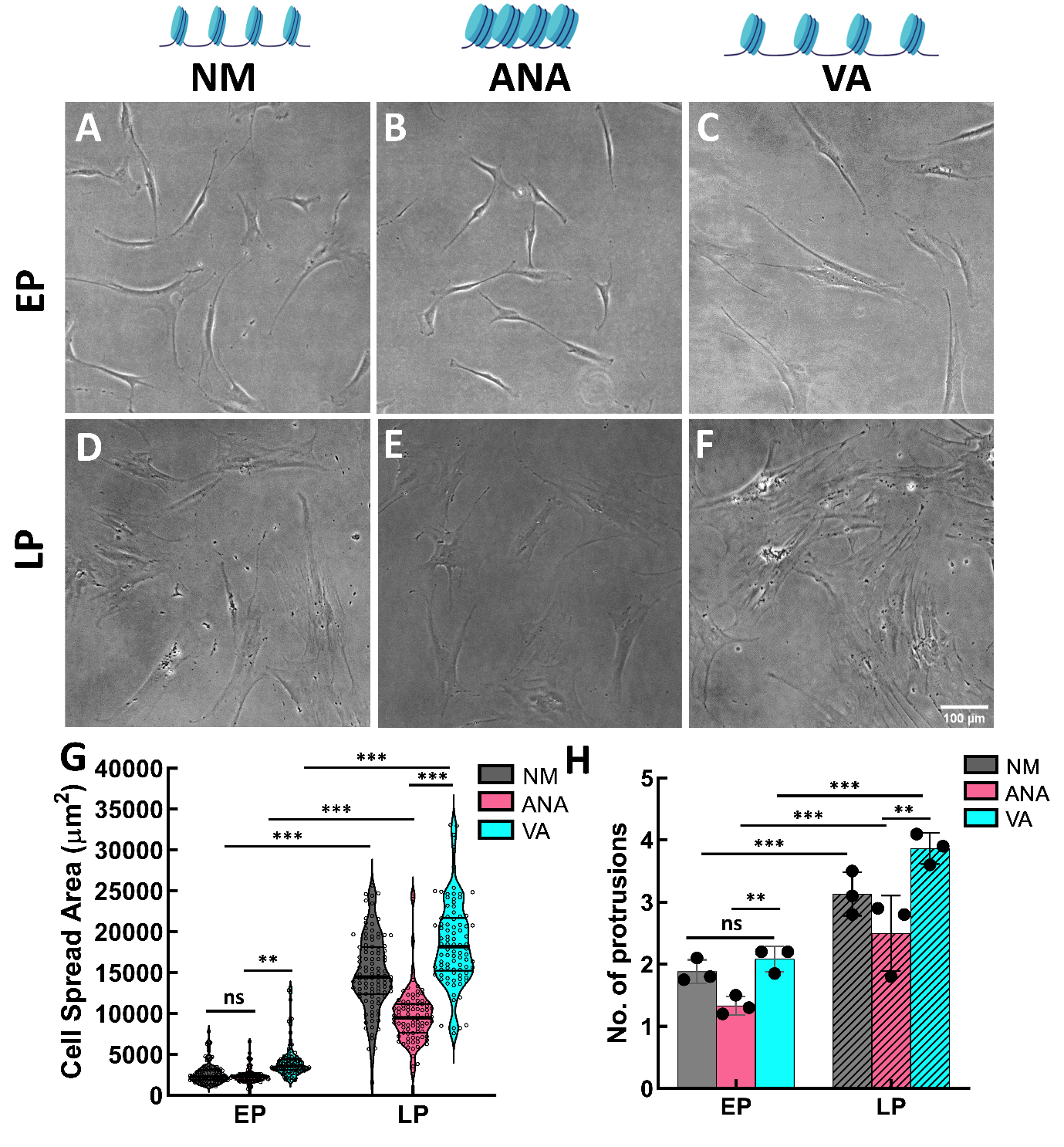


Figure S1. Representative phase contrast images of hMSCs cultured in early passage cells in (A) ctrl (NM), (B) presence of ANA and (C) presence of VA and in late passage cells in (D) ctrl (NM), (E) presence of ANA and (F) presence of VA; (G) Graph comparing cell spread area between early and late passage cells in all three conditions between early and late passage cells ; (H) graph comparing number of cellular protrusions in all three conditions between early and late passage cells (NM- normal media, ANA- anacardic acid, VA – valproic acid); (n> 100 cells, 3 independent samples, Scale bar = 100 µm *p<0.05, **p<0.01, ***p<0.001, ns = non-significant).


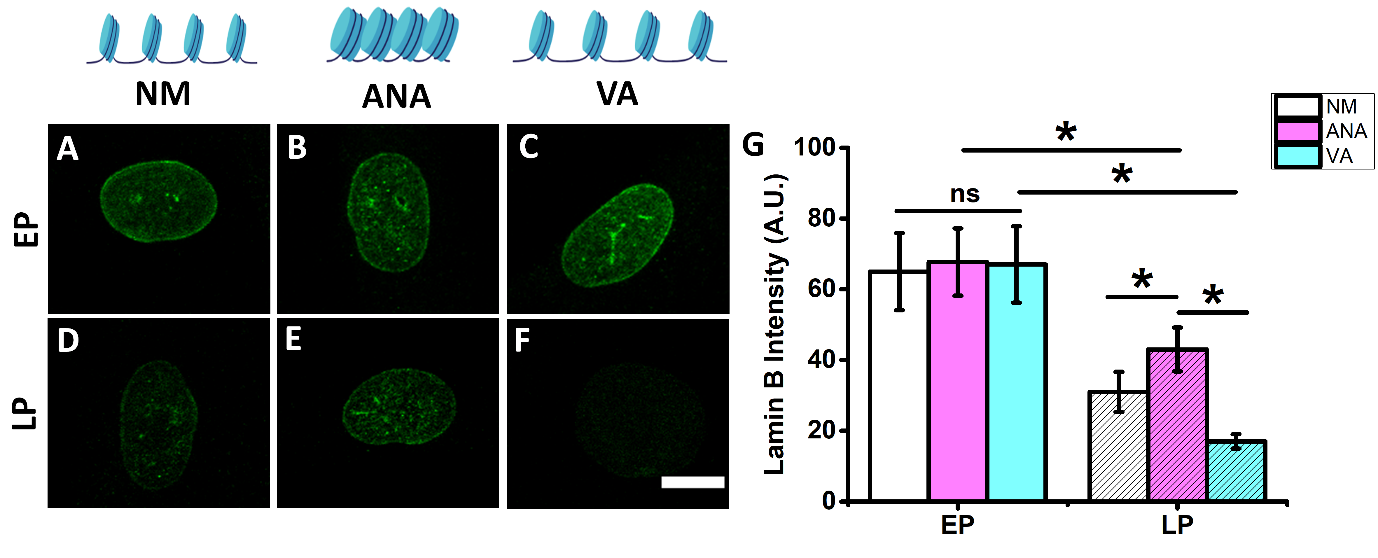


Figure S2. Immunofluorescence images of lamin B in early passage (EP, A-C) and late passage (LP, D-F) for hMSCs cultured in normal media (NM, A and D), with anacardic acid (ANA, B and E), and with valproic acid (VA, C and F). (G) Graph comparing lamin B intensity under various conditions (NM, ANA, VA), (NM- normal media, ANA- anacardic acid, VA – valproic acid); (n> 25 nuclei, 2 independent samples, Scale bar = 10 µm, *p<0.05, ns = non-significant).


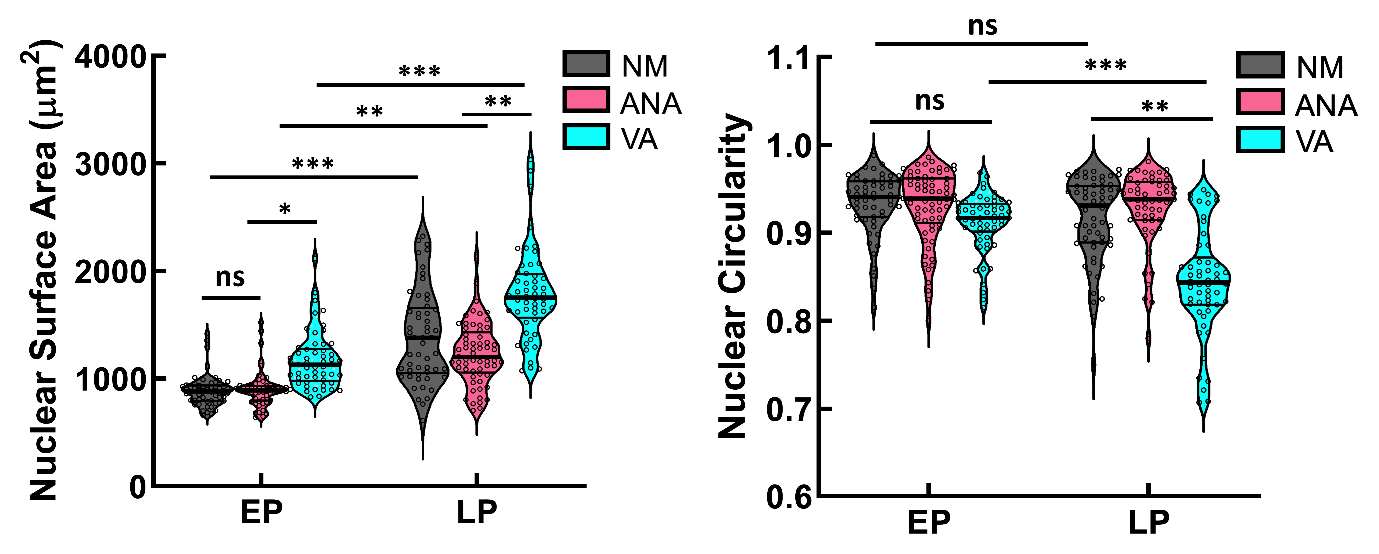


Figure S3. Graph comparing nuclear surface area and nuclear circularity under various conditions (NM- normal media, ANA- anacardic acid, VA- valproic acid) between early and late passage (n> 50 nuclei, 3 independent samples, Scale bar = 10 µm, *p<0.05, *p<0.05, **p<0.01, ***p<0.001, ns = non-significant).
